# Comparison of conventional and extended middle meatal antrostomy for the treatment of antrochoanal polyps

**DOI:** 10.1186/s12893-023-01918-7

**Published:** 2023-01-21

**Authors:** Huiqin Zong, Zhengcai Lou

**Affiliations:** Department of Otorhinolaryngology, Wenzhou Medical University Affiliated Yiwu Hospital, 699 Jiangdong Road, Yiwu, 322000 Zhejiang China

**Keywords:** Antrochoanal polyps, Endoscopic sinus surgery, Middle meatal antrostomy, Recurrence

## Abstract

**Objective:**

The objective was to compare the recurrence rate and complications between endoscopic middle meatal antrostomy (MMA) and extended MMA for the treatment of antrochoanal polyps (ACPs).

**Methods and materials:**

95 ACPs were prospectively enrolled to undergo MMA (n = 48) or extended MMA (n = 47). The recurrence rate and complications were compared between these groups.

**Results:**

All patients completed 24 months of follow-up. The recurrence of ACP was demonstrated by only endoscope at 6 and 12 months postoperatively but at 24 months postoperatively by endoscope and CT. Rates of recurrence of ACP differed between groups and significance at postoperative month 6 (7/48 patients in the MMA group and 0/47 patients in the extended MMA group, *P* = 0.02), month 12 (16/48 vs. 2/47, *P* < 0.01) and month 24 (21/48 vs. 3/47, *P* < 0.01). No MMA closure was found in any group, 19.15% (9/47) patients complained of cheek numbness in the extended MMA group, however, no major complications were observed in both groups.

**Conclusions:**

Extended MMA via antidromic extended medial wall of MS may effectively reduce the recurrence of ACP with lower complications.

## Introduction

Antrochoanal polyps (ACPs) are seen in young and children, which arises from the maxillary sinus (MS) to reach the ipsilateral choana [1]. In the past, it was usually treated by nasal polypectomy or Caldwell–Luc (CWL) approach, which resulted in the high recurrences [1]. With the development of endoscopic sinus surgery (ESS), middle meatal antrostomy (MMA) has been widely used to treat the ACPs [1–3]. Nevertheless, MMA was not always possible to locate and reach the maxillary stalk, especially on the anterior or inferior wall [4], ESS combined approach had been used to reduce the risk of recurrence, including CWL procedure [5], transcanine sinusoscopy [1], mini-CWL [6], and inferior meatus antrostomy (IMA) [4]. Although these techniques had been applied, recurrences and complications were still high in children [1]. In recent years, extended MMA was introduced to treat the ACPs in our department, with the low recurrence rate and complications. The objective of this study was to compare the recurrence rate and complications between MMA and EMMA for the treatment of ACPs.

## Materials and methods

This was a prospective cases controlled trial. Study subjects were recruited from consecutive patients listed for surgery for ACPs who visited the Department of Otorhinolaryngology, Head and Neck Surgery, between March 2011 and January 2018. Cases that met the following criteria were included: unilateral ACPs, no obvious bone destruction (except the absorption of extruding bone) on computed tomography (CT). The exclusion criteria were as follows: involvement of another nasal sinus on CT, histological evidence of a malignant tumor, chronic sinusitis or odontogenic sinusitis, and revision surgery. The patient age, sex, duration of symptom, lesion side, as well as the surgical procedure(s) performed, recurrence, complications, and follow-up duration were recorded. Participants with an odd number in the mantissa of their registration number were allocated to a MMA and those with an even number to an Extended MMA.

### Surgical procedure

All patients were placed supine with the head slightly elevated; hypotensive general anesthesia was then induced. Cotton mixed with decongestants was inserted into the nose 10 min before surgery. All procedures were performed by experienced surgeons using rigid 0°, 30°, or 70° 4-mm endoscopes. No patient was treated via the CWL approach.

### MMA

MMA technique has been described by previous authors [4, 5]. The uncinate process was resected following debridement of nasal parts of polyps, and identification and enlargement of the natural sinus ostium. The MMA was enoughly enlarged to facilitate access to the maxillary region. After satisfactory endoscopic visualization, the polyps were debulked with a microdebrider to further identify the origin, and the underlying mucosa was removed with preservation of the normal mucosa.

### Extended MMA

The uncinate process was resected following debridement of nasal parts of polyps, and a wide MMA was created by removing most of the posterior fontanelle and connecting a possible secondary maxillary ostium to the area of the maxillary natural ostium anteriorly. In addition, when the polyp was located on the anterior, inferior medial, or inferior side of the MS, the MMA was further enlarged via extended medial wall using drilling of bone and reverse bone rongeur, which was similar to the partial medial maxillectomy with preservation of the inferior turbinate and but didn’t injury the nasolacrimal duct (NLD). The retrogradation removal of medial mucosa was performed to clearly expose the wall of MS. After widening of the antrostomy, attachment of ACP was identified and completely removed using curved microdebrider blades. Normal MS mucosa should be reserved.

### Postoperative management

None of patients underwent surgery of septum and the turbinates, while all patients applied nasal packing with Merocel [Medtronic Xomed, Jacksonville, Fla.] to prevent postoperative bleeding, and all specimens were sent to the Department of Pathology. Nasal packing was removed on the 2nd postoperative day; daily saline nasal spray was then performed. Topical or systemic steroids (methylprednisolone, 20 mg per day) were prescribed if mucosa edema was detected during the first follow-up visit (1 month after surgery). Postoperative follow-up endoscopy was easily scheduled 1, 6, 12, and 24 months after surgery for all patients. In addition, postoperative CT was performed 24 months after surgery to accurately assess the recurrence of ACP.

### Statistical analysis

Statistical analysis was performed using the Mann–Whitney U test or Fisher’s exact test. A P-value of < 0.01 was considered statistically significant.

## Results

### Demographic data

Total of 95 with ACPs were included in the analysis, all patients completed the follow-up of 24 months. Of the 95 patients, 48 patients were in the MMA group and 47 in the extended MMA group, the average age, sex, duration, side, and follow-up time were matched among the groups (Table 1).

**Table 1:** Demographic data among two groups

	MMA (n = 48)	Extended MMA (n = 47)	P value
Average age, years	17.4 ± 2.49	18.9 ± 3.47	0.845
Sex (female vs male)	36:12	33:14	0.769
Side (L:R)	42:6	40:7	0.967
Duration of symptom, years	9.31 ± 1.76	10.11 ± 0.85	0.713
Average operation time, minutes	37.61 ± 5.42	48.64 ± 5.42	< 0.01
Follow-up time, years	25.21 ± 1.39	26.03 ± 1.92	0.861

The site of origin was not identified in 15 (15.79%), but was identified as the inferomedial wall in 34 (35.79%) patients, the inferolateral wall in 22 (23.16%), the inferior wall in 14 (14.74%), the anterior wall in 10 (10.53%). In addition, natural sinus ostium were naturally enlarged because of extrusion of ACPs compared to contralateral ostium in all the patients.

### Surgical outcome

**Fig. 1:**
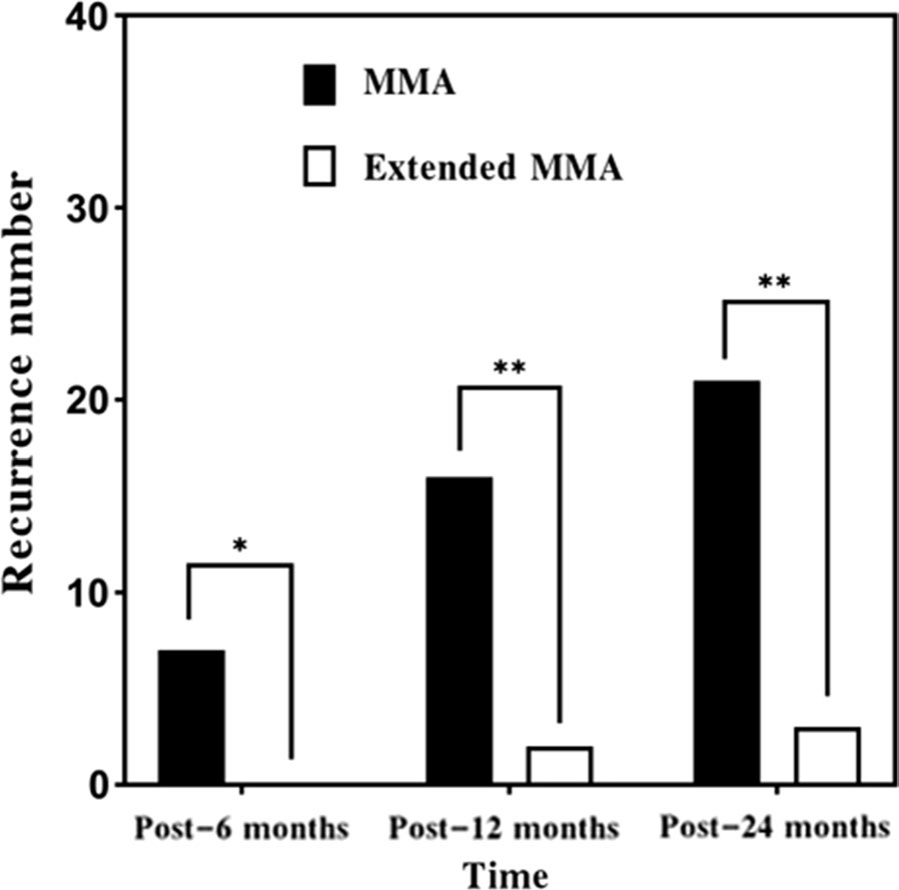
The recurrence numbers of patients among two groups at different follow-up points

**Fig. 2:**
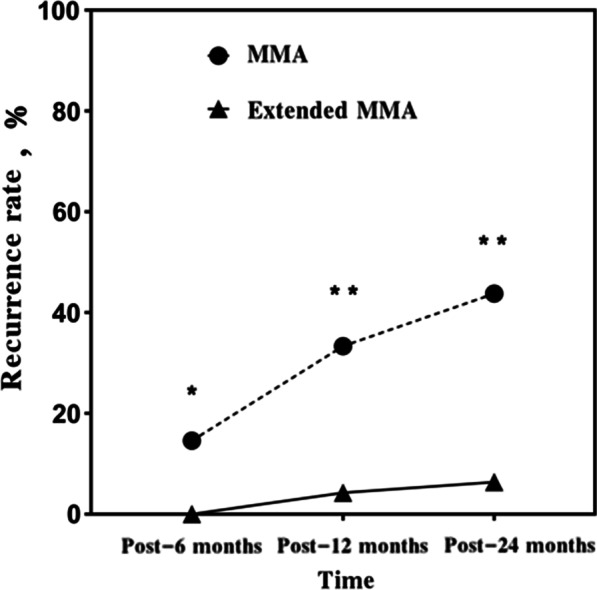
The percentage of ACP recurrence among two groups at different follow-up points

**Fig. 3:**
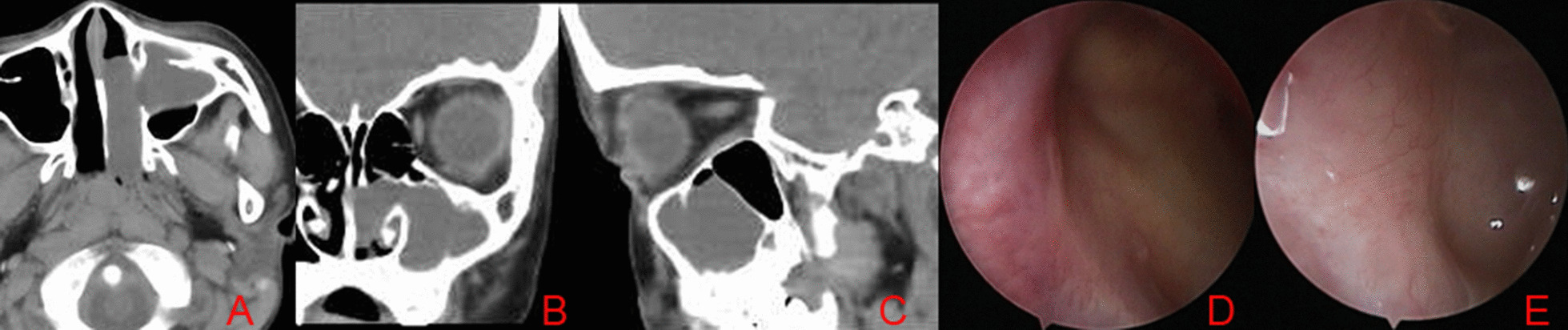
Preoperative CT revealed left ACP (**A**–**C**) in the patients with extended MMA, the maxillary sinus cavity via MMA at 6 months postoperatively (**D**) and well epithelization at 12 months postoperatively (**E**)

The endoscopic recurrence rate was summarized at different follow-up points in the Table 2, Figs. 1, and 2. The recurrence of ACP was demonstrated by only endoscope at 6 and 12 months postoperatively but at 24 months postoperatively by endoscope and CT. Rates of recurrence of ACP differed between groups and significance at postoperative month 6 (7/48 patients in the MMA group and 0/47 patients in the extended MMA group, *P* = 0.02), month 12 (16/48 vs. 2/47, *P* < 0.01) and month 24 (21/48 vs. 3/47, *P* < 0.01). Figure 3 showed endoscopically no recurrence in 9 years male with left ACP at 12 months postoperatively.

**Table 2:** Postoperative recurrence rate at 6-, 12-, and 24 months among two groups

	MMA (n = 48)	Extended MMA (n = 47)	*P*
Post-6 months, n (%)	7 (14.58)	0 (0.00)	0.02
Post-12 months, n (%)	16 (33.33)	2 (4.26)	< 0.01
Post-24 months, n (%)	21 (43.758)	3 (6.38)	< 0.01

For the most of recurrent cases in both groups, revision surgery was performed through the extended MMA in the MMA group and by first enlarged MMA opening in the extended MMA group; but revision surgery was rejected in 7 patients in the MMA group. No MMA closure was found in any group but the opening had become smaller in the MMA group after 24 months in some patients. In addition, 19.15% (9/47) patients complained of cheek numbness in the extended MMA group, however, no major complications were observed in both groups.

## Discussion

CWL procedure was traditionally used for the treatment of ACPs, that was not only associated with a high recurrence rate, but also with complications [1]. Although MMA became the mainstay of treatment for ACPs in recent years, it didn’t view the whole MS, especially for the anterior and inferior walls, thereby also resulted in high recurrence rate [1, 4]. The identification and removal of the origin are vital for preventing the ACP recurrence. Previous studies showed that most unilateral ACPs originated from the lateral wall and inferior regions [4, 7]. Al-Balas et al. [4] reported that 31.7% were located in the inferior wall, 12.2% in the anterior wall, 9.8% in the lateral wall, and 4.9% in the posterior wall. El-Sharkawy [8] reported that 38.9% were located in the posterior and lateral walls, 36.1% in the medial wall, and 8.3% in the inferior wall. Some scholars found that 7/12 were in the inferolateral wall, while 2/12 were in the inferomedial and superomedial walls, and 1/12 were in the margin of the ostium [9]. In this study, the ACP was located in the inferomedial wall in 35.79%, in the inferolateral wall in 23.16%, in the inferior wall in 14.74%, and in the anterior wall in 10.53%. However, rigid endoscope and instrument didn’t reach all corners of MS via traditional MMA.

In this study, we performed a extended MMA via retrograde removal of medial wall to remove the ACP. The endoscopic recurrence rate in the extended MMA group was significantly lower at any follow-up point compared with MMA alone group. The extended MMA clearly exposed all the wall of MS, especially for the antero inferior portion, which was similar to standard MMA combined with IMA. The extended range of was based on the origin of ACP. Although recent some scholars recommended MMA combined with IMA or CWL procedure to reduce the recurrence [4, 5], the recurrence rate in the extended MMA in this study was similar with these scholar’s findings [4, 5, 10, 11]. These scholars reported the recurrence rate of 3% using MMA combining IMA [4], 12.5% using MMA plus the CWL [5], 21.05% using MMA combined with the mini-CWL [11], 5.3% using MMA combined with the CWL [5]. Thus, the extended MMA procedure was comparable to previous combining approach.

Surgical complications should be considered for paediatric patients. CWL procedure resulted in postoperative cheek bulging, cheek pain, dental problems, etc. [1]. However, IMA easily damaged the ductus nasolacrimalis, thereby caused epiphora and closure of IMA antrostomy [4]. CWL and IMA also changed the drainage and ventilation of the MS [12]. In addition, even if angled endoscope was applied, it was sometimes difficult to visualize the site of origin of ACP in the MMA group, which resulted in the possibility of residual ACP, thereby increased the recurrence. On the contrary, the site of origin of ACP could be well identified in the extended MMA procedure, nevertheless, the extended MMA had the limit of anterior extension. No major complications were observed in both groups in this study.

## Conclusions

Extended MMA via antidromic extended medial wall of MS may effectively reduce the recurrence of ACP with lower complications.

## Data Availability

All data generated or analyzed during this study are included in the published article.
